# The neural response properties and cortical organization of a rapidly adapting muscle sensory group response that overlaps with the frequencies that elicit the kinesthetic illusion

**DOI:** 10.1371/journal.pone.0188559

**Published:** 2017-11-28

**Authors:** Paul D. Marasco, Dennis J. Bourbeau, Courtney E. Shell, Rafael Granja-Vazquez, Jason G. Ina

**Affiliations:** 1 Advanced Platform Technology Center of Excellence, Louis Stokes Cleveland Department of Veterans Affairs Medical Center, Cleveland, Ohio, United States of America; 2 Laboratory for Bionic Integration, Department of Biomedical Engineering, Lerner Research Institute, Cleveland Clinic, Cleveland, Ohio, United States of America; 3 Functional Electrical Stimulation Center, Louis Stokes Cleveland Department of Veterans Affairs Medical Center, Cleveland, Ohio, United States of America; 4 Department of Physical Medicine and Rehabilitation, MetroHealth Medical Center, Cleveland, Ohio, United States of America; 5 Research Service, Louis Stokes Cleveland Department of Veterans Affairs Medical Center, Cleveland, Ohio, United States of America; University of Chicago, UNITED STATES

## Abstract

Kinesthesia is the sense of limb movement. It is fundamental to efficient motor control, yet its neurophysiological components remain poorly understood. The contributions of primary muscle spindles and cutaneous afferents to the kinesthetic sense have been well studied; however, potential contributions from muscle sensory group responses that are different than the muscle spindles have not been ruled out. Electrophysiological recordings in peripheral nerves and brains of male Sprague Dawley rats with a degloved forelimb preparation provide evidence of a rapidly adapting muscle sensory group response that overlaps with vibratory inputs known to generate illusionary perceptions of limb movement in humans (kinesthetic illusion). This group was characteristically distinct from type Ia muscle spindle fibers, the receptor historically attributed to limb movement sensation, suggesting that type Ia muscle spindle fibers may not be the sole carrier of kinesthetic information. The sensory-neural structure of muscles is complex and there are a number of possible sources for this response group; with Golgi tendon organs being the most likely candidate. The rapidly adapting muscle sensory group response projected to proprioceptive brain regions, the rodent homolog of cortical area 3a and the second somatosensory area (S2), with similar adaption and frequency response profiles between the brain and peripheral nerves. Their representational organization was muscle-specific (myocentric) and magnified for proximal and multi-articulate limb joints. Projection to proprioceptive brain areas, myocentric representational magnification of muscles prone to movement error, overlap with illusionary vibrational input, and resonant frequencies of volitional motor unit contraction suggest that this group response may be involved with limb movement processing.

## Introduction

Proprioception is the sense of one’s position, orientation, and movement in space. Kinesthesia is a component of proprioception and relates to the sense of limb movement. Kinesthesia is fundamental to efficient motor control as disruptions to this sense result in crippling functional deficits. The loss of only proprioceptive feedback, such as in large-fiber sensory neuropathies, completely disrupts limb and postural control even when the motor system is functionally intact, leaving the individual unable to maintain posture or move in any meaningful way when vision is occluded [1]. Complications of kinesthetic integration also play a role in movement disorders such as in Parkinson’s and Huntington’s diseases [2].

The neural substrates and organizational properties of kinesthesia are complex and remain poorly understood. There appear to be many contributors to the perception of limb movement. In the central nervous system there is evidence that the sensory cortex is activated during limb movement [3–5], and that during passive limb movement the motor cortex is activated as well [4–6]. In the peripheral nervous system, it is known that both active and passive limb movements as well as joint position are signaled through a number of different afferent receptors. These receptor types include primary muscle spindle fibers [7–9] and joint capsule receptors [7]. While it is considered unlikely that Golgi tendon organs or secondary muscle spindle afferents are involved [10] other investigators have observed projections from Ib afferents [11] and could not rule out the contributions of these afferent types [3, 11, 12]. Cutaneous mechanoreceptors associated with joints also signal limb position such as RA-1 units and SA-I and SA-II units [13]. Further, it appears that both deep afferent receptors and cutaneous receptors contribute to sensation of limb position [9, 10, 14]. Nonetheless, muscle spindle fibers are widely considered to be the candidates that contribute predominantly to the sensation of limb movement and direction [7, 8, 15–19]. Yet while there appear to be many aspects to limb movement sensation, establishment of an organizational framework for this sense has remained elusive.

Much of what is known about the properties of limb movement sensation comes from the kinesthetic illusion. In humans, vibrating the tendon of a limb muscle at frequencies between 70 and 115 Hz generates a strong joint-specific perception that the limb is moving even though it is actually immobile [15, 20]. Coupling this approach with brain imaging has led to insight into determining the brain areas that are active during kinesthetic perception [21–24]. However, the lack of separation between cutaneous and muscle sensory inputs prevents defining the roles of these distinct modalities, and the lower resolution of earlier imaging studies prevents resolving many of the detailed organizational properties. As such, a clear pathway of kinesthetic sensibility has not been well established.

Here we use the mechanical vibrational input of the kinesthetic illusion and electrophysiological approaches in rats to explore the peripheral activity and cortical organization of a muscle sensory group response. We provide evidence of a rapidly adapting type sensory group response with electrophysiological properties that are distinct from type Ia muscle spindle fibers. This RA-MS-type group response is tuned to the vibrational frequency that triggers the kinesthetic illusion in humans. This group appears to communicate input to a discrete brain area that is separate from the histologically defined cutaneous mechanosensory forepaw and shoulder representation in primary somatosensory area (S1). The representational organization of this region of cortex is muscle-specific (myocentric) and magnified with respect to proximal and multi-articulate limb joints. These data suggest that, in addition to muscle spindle and cutaneous sensory inputs, this RA-MS-type group response may be involved with limb movement sensation.

## Materials and methods

### Animal model

Neuronal activity in response to the same mechanical perturbations was recorded from both forelimb peripheral nerves in 15 animals, and from cortical recordings in 7 separate animals. Male Sprague Dawley rats (Envigo, East Millstone, NJ) were used in these experiments because of their well-developed and well-characterized somatosensory cortical limb representation [25–28]. In addition, these animals use their forelimbs for dexterous manipulation in a manner similar to humans [29, 30]. All procedures were approved by the Institutional Animal Care and Use Committee of the Louis Stokes Cleveland Department of Veterans Affairs Medical Center.

### Peripheral nerve electrophysiology and mechanical stimulus parameters

For the surgical preparation the animals were initially induced to an anesthetic plane with 2.5% isoflurane in 100% O_2_ (VetEquip, Pleasanton, CA), then dosed with a single IP bolus of 30% urethane at 1.5g/kg and removed from gas anesthesia. The surgical plane was maintained early in the procedure with supplemental IP doses of 22.5 mg/kg ketamine hydrochloride and 3 mg/kg xylazine. Body temperature was maintained with a water-circulating heating pad (Gaymar, Braintree, MA). Hemostasis was maintained with electrocautery (Bovie Medical, FL). The right forelimb was degloved to silence input from the forelimb cutaneous receptors. A ring incision was made at the interface between the glabrous palm and wrist around the back of the forepaw to the metacarpophalangeal joints, and the forelimb was then completely degloved towards the shoulder. The distal median nerve and branches were ligated and cut at the wrist. The individual forelimb muscles and distal tendons of the anterior compartment were blunt-dissected free from origin to wrist. Care was taken to maintain the integrity of the median nerve branches to the forelimb muscles. The proximal median nerve trunk was dissected free at the humeral level and sectioned near the axilla. The proximal fibers of the nerve stump were teased on to a piece of glass coverslip. A pool of warmed saline was created over the teased nerve with a vacuum grease dam.

Peripheral nerve recording experiments were performed in 15 animals (average weight 457.4 g, SD 68.1). A 65 kΩ 51 μm diameter PFA-insulated stainless steel “type 316 composition” wire hook electrode (A-M Systems, Carlsborg, WA) was used to record single axon action potentials at the proximal median nerve stump. The resulting neural signals were amplified (Bak Electronics, Inc. Model A-1, Umatilla, FL) and passed to an oscilloscope (BK Precision 2120B, Yorba Linda, CA), filters and audio speaker (Neurolog 125, 126, and 120, Digitimer, Ft. Lauderdale, FL), and recorded with a Cambridge Electronic Design (CED) Power 1401 computer interface (Cambridge, UK) sampling at 83.3 kHz. The spikes from the stimulus presentations (described below) were counted and sorted (as needed to differentiate between clearly separate axon signals [no more than two] on a minority of recording traces) using Spike 2 software (Cambridge, UK).

While listening to the single unit audio output from the speaker, the search for neural activity was conducted by slowly pulling the forelimb tendons with forceps, sharply tapping the forceps with a padded probe, and by vibrating the tendons at 70 Hz with 1mm displacement (peak-to-trough). Vibration of the tendons was done using a blunt probe mounted on a position-controlled voice coil sliding stage linear motor with a 150 nm resolution optical linear displacement sensor (Equipment Solutions, VCS-10, SCA-824, Sunnyvale, CA). This vibration was undertaken to provide input to the tendon and muscle that reflected the kinesthetic illusion [20].

Upon isolation of a well-defined single unit response that mapped to a single forelimb muscle, the axonal responses were recorded time locked with the position sensor output of the voice coil stage linear motor, which was then mounted to an adjustable armature, and the blunt probe was replaced with a small clip and attached to the freed distal tendon. The clip was retracted to the point just where slack was taken from the muscle. Then slight tension was applied to stretch the muscle, equal to but not exceeding 20% of the measured length (origin to myotendinous junction). With this slight tension in the muscle the following stimulus paradigms were applied: an initial 2 mm stretch at approximately 150 mm/s followed by a 3 mm/s ramp-and-hold repeated 5 times, and sinusoidal vibrational input starting at 0 mm at 1, 3, 6, 10, 50, 60, 70, 80, 90, 100 Hz at 100, 250, 500, 1000 μm displacements for 100 cycles repeated 3 times each. Responses to vibration that resulted in a spike for every sinusoidal cycle (at least 98 but not over 102 spikes for each of the 100 sinusoidal cycles) were considered 1:1 [31]. Average spikes/impulses per vibration cycle were calculated at all frequencies and displacements. Tonic square wave response profiles were measured with 2 mm displacements for 250 ms and 500 ms, 36 times each.

### Cortical electrophysiology and mapping

Cortical recording experiments were performed in 7 animals (average weight 436.6 g, SD 99.7) using the identical surgical preparation, stimulus paradigms, signal recording, and spike sorting as described above for peripheral recordings. Briefly, the right forelimb was degloved and the musculature was separated by blunt dissection (as described above). The forelimb was then wrapped with gauze and kept moist with saline. Instead of recording from the peripheral median nerve, a craniotomy was performed over the contralateral hemisphere and silicon oil was used to cover the brain. A macro photograph was taken of the cortical surface, enlarged, and used as a guide to mark electrode penetrations. Multiunit recordings were made with a single 1.0 MΩ tungsten microelectrode (Impedance measured at 1 kHz, Microprobes, Gaithersburg, MD) 700 μm deep to the pial surface of the cortex. We made 524 electrode penetrations across the left cortex of the 7 animals with an average of 75 penetrations for each animal. All cortical responses were amplified and passed to audio as described above.

For the first phase of the mapping experiments the distal branches of the median, radial, and ulnar nerves that served the glabrous and distal skin of the forepaw were marked with loops of 6–0 monofilament suture and left intact. These were used to verify the viability of the forelimb nerves following the surgical degloving and muscle separation procedures and to help determine the general location of the primary somatosensory cortical (S1) body representation. While listening to the audio output of multiunit activity over the speaker, the S1 cutaneous body representation was mapped by placing the electrode in the brain at various points and by confirming the activity at these locations while applying a mechanical stimulus with a cotton-tipped probe to the contralateral body surface including: whiskers, buccal pad, glabrous forepaw, dorsal digits, hindpaw, and body. The location of each penetration was marked on the photograph of the cortical surface and the receptive fields for each of the penetrations were then drawn on schematic diagrams of the rat skin surface. Areas of no response were also noted.

Once a general layout of the S1 body representation had been established, phase two of the experiment began where the previously marked nerve branches (median, ulnar, and radial) that served the remaining skin of the distal forepaw were ligated and cut to silence the input from any remaining cutaneous receptors. Cortical recording was resumed and electrode penetrations were placed randomly across the left brain surface. Receptive fields at every penetration were delineated by first brushing the body surface with the handheld probe to check for cutaneous mechanosensory responses, and then grasping each of the individual distal tendons of the forelimb with forceps and tapping the muscle sharply in line with the muscle to elicit responses from its sensory receptors. Any cutaneous receptive fields were drawn on the rat skin surface diagrams. Penetration points that were responsive to tapping the individual muscles of the forelimb were marked and the receptive fields were drawn on separate schematics of the rat forelimb musculature. At each cortical electrode penetration point that was responsive to forelimb muscle all distal tendons of all of the isolated muscles of the forelimb were tapped in succession to verify the number of individual muscles that showed receptive fields at that specific point. Determinations of names of the specific muscles were made by comparing across a rat-specific and a human anatomical atlas [32, 33]. Once a muscle sensory response was identified, adjacent points were probed in the local area to delineate the scope and location of any related responses. Non-responsive sites were noted; penetrations in the auditory cortex were verified by hand claps; penetrations in the visual cortex were verified by flashlight and shadowing the eyes from the surgical microscope.

When a strong muscle-specific response of any kind was elicited from tapping a forelimb tendon the receptive field was delineated and the distal end of the tendon was then attached to the voice coil stage linear motor. The cortical multiunit spikes were recorded on the CED unit while the identical ramp-and-hold, sinusoidal vibrational, and tonic stimulus paradigms described in the peripheral recording methods were applied.

In addition to running these stimulus inputs in the cortex we also added an additional test of velocity sensitivity where the muscles were stretched across a series ramp-and-hold stretches at 2, 10, 15, 30, 45, 70, 110, 150 mm/s, with each stretch repeated 5 times. At the end of cortical recording electrolytic lesions were placed in the cortex to use as marking points for aligning the cortical histology to the electrode penetrations.

During the sinusoidal and velocity sensitivity trials we found that the intrinsic spontaneous cortical activity made it difficult to reliably analyze data based solely on sorted spike data. In these two situations we relied on signal power to determine cortical activity characteristics. The signal power in relation to stimulus inputs was calculated in the first 100 ms of the cortical signal following the onset of the mechanical stimulus as:

$$Signal\;Power=\frac{\int_{t0}^{t1}\left|V\left(t\right)\right|*dt}{t1-t0}$$

Where *t0* and *t1* represent time zero and 100 ms following stimulation, respectively, and *V(t)* represents the cortical signal voltage. The power in the signal in absence of stimulation was subtracted and this corrected signal power was averaged across the repetitions of each test, and then normalized by the maximum signal power observed for that cortical channel. Power in the cortical signal was considered significant if it was greater than the mean plus two standard deviations of the baseline.

Latencies to cortex were measured by taking the average time to first spike following a square wave displacement for each muscle sensory peripheral unit, muscle sensory cortical unit, and for a Pacinian mechanoreceptor in the skin recorded in the cutaneous representation of S1. The propagation delay from periphery to cortex was calculated by subtracting the average latency for the peripheral units from the average latency for each of the cortical populations. Units were excluded (1 peripheral and 1 cortical) that occurred outside of the 6.8 ms physical movement of the stimulator movement as measured from the first latency for each population.

We used averaged peristimulus time histograms (PSTHs) from 10 RA-MS-type peripheral single units and 22 SA (6 Type Ia, and 16 Type II) peripheral single units to serve as comparison templates against PSTHs of spike-sorted cortical single unit recordings. During applied ramp-and-hold stimuli (n = 20), cortical PSTHs were averaged across 5 stimulus applications. Cortical PSTHs during square wave stimuli (n = 28) were averaged across 36 stimulus applications. We did not have a commensurate square wave dataset from the peripheral recordings so we constructed evaluation PSTH templates from raw data using the square wave portions of the peripheral ramp-and-hold stimuli. Data for the square wave PSTH templates took the spike count from 0.25 s before the first step up, 0.25 s of the static hold after the step up, and 0.25 s following the last step down. Using a bin size optimization method described by others [34] we optimized the PSTH bin size for all comparisons to 10 ms in order to maintain the underlying firing profiles of all three groups of recordings (RA-MS-type peripheral, SA peripheral, and all cortical).

We assessed the response properties during ramp-and-hold and square wave stimuli with two complementary approaches: PSTH-based classification and Pearson’s correlations. The PSTH-based classification approach identified each single-unit cortical PSTH as either RA-MS-type or SA based on the peripheral template PSTH to which it was closest, as determined by the shortest Euclidean distance [35]. We calculated Pearson’s correlations to describe the similarity between the PSTH for each cortical unit recording and the peripheral template PSTHs.

### Histology and image processing

At the termination of brain recording the animals were euthanized with an IP dose of 100 mg/kg sodium pentobarbital and perfused transcardially with 1x phosphate buffer saline (PBS) pH 7.4 followed by 4% paraformaldehyde (PFA) in PBS. The cortical hemispheres were removed, post-fixed overnight in 4% PFA, and then cryoprotected for 24 h in 30% sucrose (in PBS). The cortex was flattened on the frozen stage of a sliding microtome (Leica SM 2000, Leica Biosystems Inc. Buffalo Grove, IL) and sectioned at 60 μm parallel to the pial surface. The sections were processed for cytochrome oxidase, known to stain tissue that is metabolically active [36] and mounted on glass slides (Brain Research Laboratories, Newton, MA). The sections showing clear staining for the whisker barrel subfields and other features of S1 were photographed using a backlit stage (Porta-Trace, Gagne Inc., Johnson City, NY) and a Nikon DS-90 digital SLR with macro (Nikon Inc., Melville, NY). Histology images were converted to grayscale and processed for contrast and brightness in Adobe Photoshop CS5 (Adobe, San Jose, CA). These were then overlaid with scans of the photograph with marked electrode penetrations and aligned using the lesion sites in Adobe Illustrator CS5 (Adobe, San Jose, CA). Outlines of the cytochrome oxidase-delineated features of the cortex were drawn over the histological photographs, and the electrode penetration points were related to the morphological boundaries of the primary somatosensory cortex (S1).

## Results

### Peripheral nerve recordings

We recorded activity from 57 muscle sensory afferent neurons in the median nerve of the upper forelimb. By surgically degloving the forelimb and denervating the forepaw of rats, we silenced confounding input from the cutaneous receptors. We performed full ramp-and-hold and sinusoidal vibrational characterizations on 33 of the recorded responses. For the remaining 24 responses we were unable to complete full characterizations due to changes in background noise, loss of signal before all stimulus paradigms were completed, or inability to clearly sort and separate overlapping responses. These 24 recordings that did not rise to the most stringent criteria for signal clarity and completion of the full battery of stimulus inputs were considered not fully characterized and removed from the sets of data. Any units that were found to be spontaneously active without responsiveness to stimulus input were not utilized for recording.

### General classification of primary muscle sensory afferent neurons

Of the 33 fully characterized responses 11 were classified as RA due to the lack of sustained response to prolonged static displacement. The other 22 isolated responses were classified as slowly adapting due to sustained activity during prolonged static displacement. Within the additional group of 24 non-fully characterized unit recordings we could broadly classify 7 as RA (2 additional as possibly RA), 10 as SA (1 additional as possibly SA), 3 spontaneously active units that were unresponsive to stimuli, and 1 unit that was lost before the data required to make a distinction between RA and SA could be recorded.

### Characterization of peripheral slowly adapting muscle sensory units

**Fig 1:**
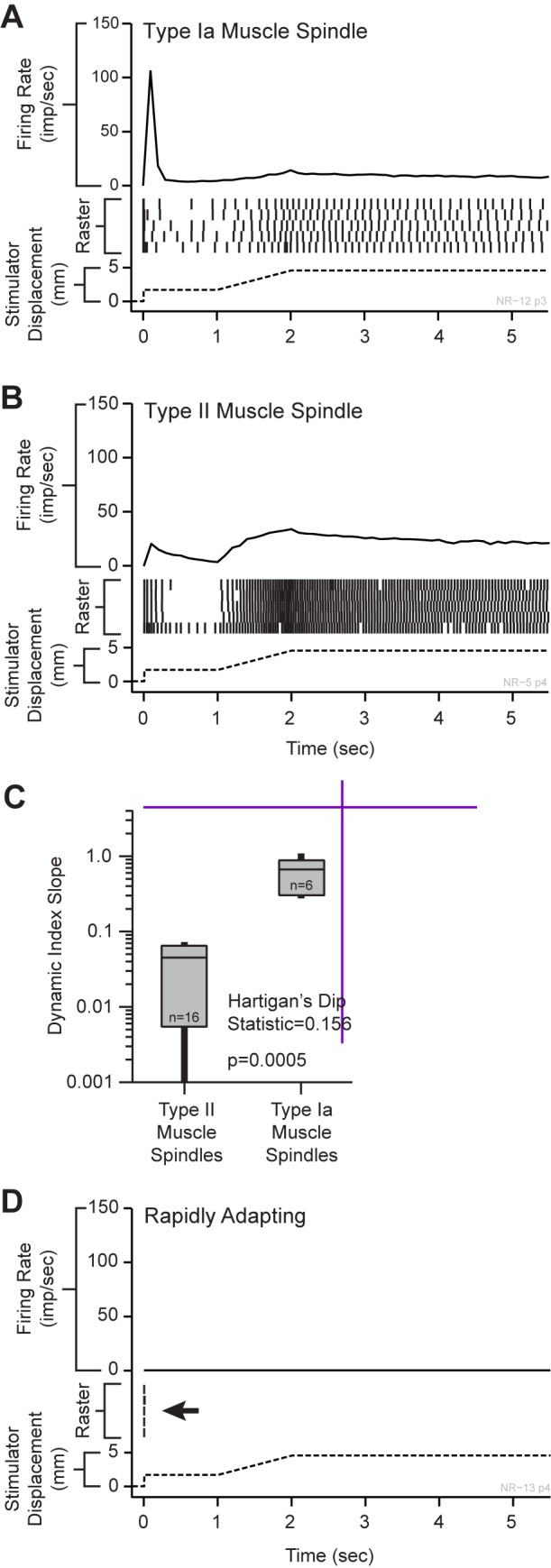
Static and dynamic response profiles for three response populations active during muscle stretch and vibrational input. **(A)** Type Ia muscle spindle fibers showed a high instantaneous firing rate for the rapid onset of the square wave input and a lower instantaneous firing rate during the 3 mm/s slower onset. These receptors discharged randomly at a strong rate during the static phase. **(B)** Type II muscle spindle fibers showed similar instantaneous firing frequencies between the square-wave onset and the 3 mm/s dynamic phases with strong and consistent activity during the static phase. **(C)** The slope of the line for dynamic index by stretch rate (dynamic index slope) between points at fast (150 mm/s) and slow (3 mm/s) stretch rates had a bimodal distribution, separating the two slowly adapting receptor classes. Type Ia muscle spindles showed slopes between 0.2 and 1.1. Type II afferents showed slopes less than 0.08. **(D)** The RA-MS response group showed a different response to the dynamic and static phases of the ramp-and-hold tendon stretching input, firing a single impulse (occasionally two) at the onset of the square wave and with silence during all other stimulus phases.

**Fig 2:**
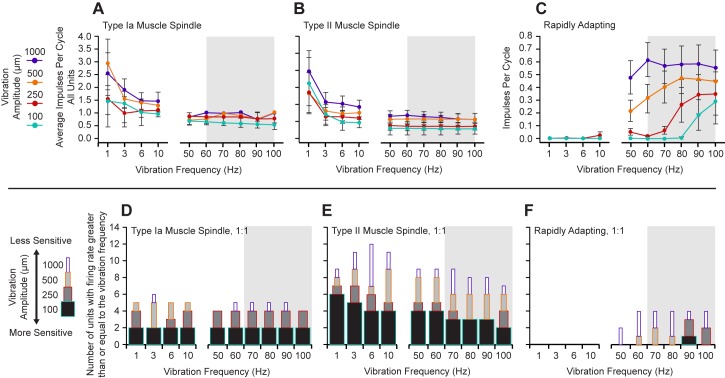
Sinusoidal displacement frequency response profiles for the three identified response groups. The grey background denotes the band of frequencies known to trigger the kinesthetic illusion [15, 40–42]. The average impulses per cycle for all of the peripheral units. **(A)** Type Ia muscle spindles displayed high average impulses per cycle across the lower frequencies. **(B)** Type II muscle spindle fibers were similar to type Ia’s in the lower frequencies as well. **(C)** The average impulses per cycle for the RA response group were higher for the measured frequencies within the active range of the kinesthetic illusion. In contrast, the type Ia and type II slowly adapting populations showed the most activity in frequencies outside of the active range for the kinesthetic illusion. 1:1 thresholds were defined as responses tracking with 98–102 spikes over 100 cycles. Vibration amplitude boxes are stacked. **(D)** Type Ia muscle spindles show a flat, yet highly sensitive (at least 1:1 at 100 μm displacements) response across all measured frequencies. **(E)** Type II muscle spindle fibers were also sensitive across the measured frequency range although more sensitive to lower frequencies. **(F)** The RA-MS-type population was specifically sensitive to the higher measured frequencies with the highest sensitivity observed at 90 Hz. The RA-MS population was mostly insensitive to the lower frequencies at all but the highest (1 mm) displacement. The most sensitive portion of the frequency response profile for the RA-MS-type group response aligns with the reported frequencies of the kinesthetic illusion [15, 40–42].

To distinguish between the different slowly adapting muscle sensory units we calculated two dynamic peaks for each slowly adapting ramp-and-hold trial, which is the difference between the maximum firing rate achieved during muscle stretch and the steady-state firing rate measured 0.5 s after muscle stretch during the hold period [37]. Each unit had the ramp-and-hold conducted 5 times, so we averaged the firing rates to calculate one pair of dynamic peaks per unit. We then used those two dynamic peaks to calculate the dynamic index for each unit. The dynamic index was defined as the difference in dynamic peaks divided by the difference in muscle stretch velocities (150 mm/s and 3 mm/s); i.e., the slope of the line between the dynamic indices plotted against the stretch rate. Hartigan’s Dip Test reveals that these slope data are bimodal (dip statistic = 0.156, p = 0.0005, slope histogram bootstrapped with 50,000 replicates) [38] and a visual inspection of the data revealed a clear gap between two groups of slope values (Fig 1C). We placed a cutoff value in this gap and used the dynamic index by stretch rate slope to differentiate between the inputs from the slowly adapting receptors, with Ia afferent responses demonstrating larger slopes than type II afferent responses [39] (see: Fig 1A and 1B). In agreement with others [39], we found a bimodal distribution of the slope between dynamic indices at different muscle stretch velocities (Fig 1C). Type Ia muscle spindle fibers maintained a steady firing rate during the static portions of the stimulus, displayed an increased firing rate when the onset velocity was high, and exhibited a lower firing rate when the onset velocity of the stimulus was lower (Fig 1A). In contrast, type II spindle fibers did not display an overtly different change in firing rate with respect to high and low stimulus onset velocities but maintained a steady firing rate during the static phase of stretch (Fig 1B). When sinusoidal frequencies from 1 to 100 Hz were applied at various amplitudes to the freed tendons, both the type Ia and type II muscle spindle populations were found to have a low frequency weighted but overall flat response tuning to all measured frequencies, with the lowest frequencies typically eliciting multiple impulses per cycle (Fig 2A and 2B). The peak 1:1 thresholds for the type Ia units were equally sensitive to the lowest displacement at all measured frequencies (Fig 2D). The type II units were also sensitive at 1:1 to the lowest displacements at all measured frequencies, although they appeared somewhat more sensitive to the lower frequencies (Fig 2E).

### Characterization of a peripheral rapidly adapting response group

**Fig 3:**
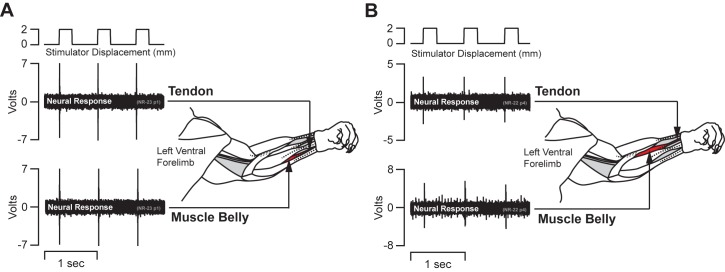
Bypassing the myotendinous junction. **(A**&**B)** Two representative single unit peripheral nerve recording traces from the median nerve with schematic drawings of the limb tendons and musculature. The neural recordings were maintained while the stimulator pulled on the freed tendon with a 2 mm displacement. The upper neural recording trace shows responses through three cycles with the stimulator pulling on the freed distal tendon. The lower trace shows three cycles of the same afferent after the stimulator was moved to the muscle belly to bypass the myotendinous junction. The nerve signal was maintained in each position.

In addition to the clearly identifiable type Ia and type II slowly adapting receptor responses, we report a population of rapidly adapting muscle sensory response profiles (RA-MS). Vibration of the freed tendons at 70 Hz resulted in the select isolation of these afferent responses. This response group readily fired a single (occasionally a second) action potential in response to the rapid onset pre-loading phase of the ramp-and-hold stretch, but were entirely unresponsive to the 3 mm/s slow ramp-up, static hold, and 3 mm/s release phases that robustly activated the type Ia and type II units (Fig 1D, arrow). The RA-MS response group was highly sensitive to a rapid pull of the distal tendon. We found that gripping the distal tendon with forceps and lightly tapping them (in line with the tendon) with a plastic probe was the most effective way to quickly and systematically isolate this group during neural recording. Similar activity with respect to similar inputs has been described in cortical recordings in area 3a of baboons [4]. Type Ia and type II response were clearly evident in peripheral recording and accounted for 67% of the total fully characterized afferents (18% type Ia and 82% type II). This RA-MS group response was also most sensitive to sinusoidal inputs in the higher frequency range with both the highest average impulses/spikes per cycle and peak 1:1 threshold activation frequencies falling within the 50–100 Hz range across the multiple displacements (Fig 2C and 2F). The average frequency across the RA-MS group for these measured responses was approximately 74 Hz. Activation for the RA-MS group at the lowest displacements was concentrated at 90 and 100 Hz. In order to investigate possible locations of these RA-MS-type units we affixed the stimulator to the distal tendon, applied a 2 mm displacement square wave for 250 ms and recorded the response. We then moved the stimulator to the muscle belly to bypass the distal myotendinous junction and applied the same square wave displacements and recorded. In all of the four different neural recordings we found that the response was activated by pulling the muscle belly without the inclusion of the distal myotendinous junction or tendon (Fig 3).

### Cortical multiunit recording and mapping

**Fig 4:**
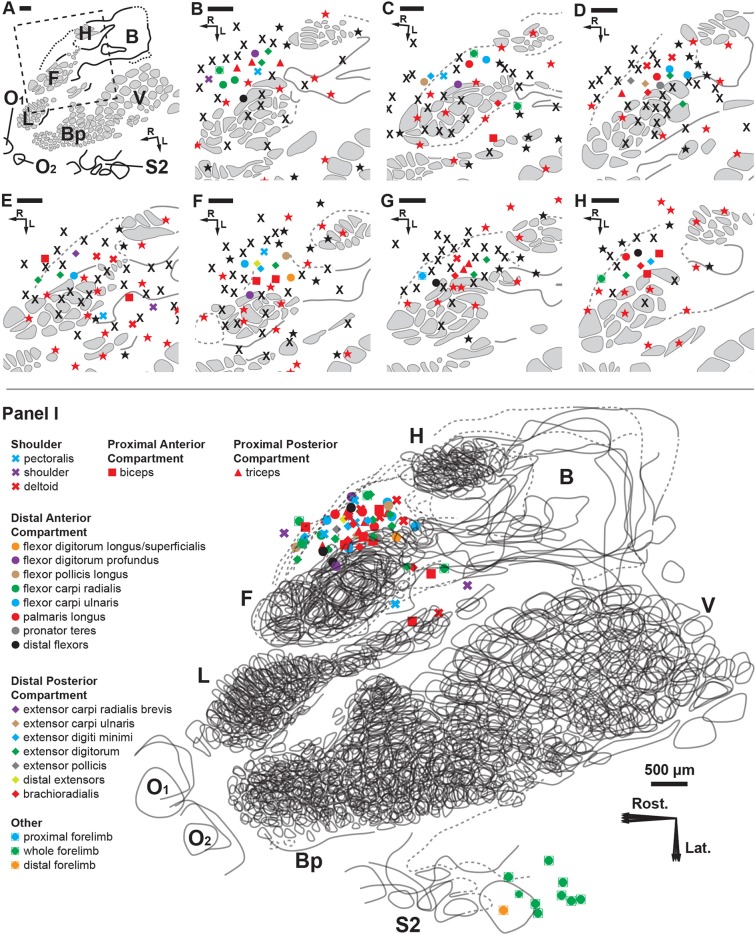
Cortical brain mapping of the RA-MS response group elicited by tapping the tendons of the degloved forelimb. Symbols represent individual electrode penetrations: **X** = No Response; **Red Stars** = Cutaneous responses **before** paw denervation; **Black Stars** = Cutaneous responses **after** paw denervation. Scale bars = 500 μm; R = rostral; L = lateral. **(A)** Overview of the left cortical hemisphere in the rat. Cytochrome oxidase delineated borders and barrel patterns of primary somatosensory cortex (S1) (black lines and grey shading): **F** = forepaw barrel subfield; **H** = hindpaw barrel subfield; **V** = vibrissae; **Bp** = buccal pad; **L** = lower lip, **O1 & O2** = oral modules 1 & 2; **L** = chin/lower lip; **B** = body; **S2** = second somatosensory area. The dashed box denotes the area of interest enlarged in **B** through **H. (B-H)** The RA-MS response group projected to a region of cortex anterior and adjacent to the forepaw barrel subfield and the area between the caudal lower lip and forelimb representations. Recordings at each electrode penetration typically yielded responses from only a single muscle of the forelimb. The RA-MS responses were entirely exclusive from the cytochrome oxidase defined borders of the somatotopic S1 cutaneous representation. **(I)** A scaled composite overlay of all seven recording cases showing the global organization of the muscle-specific tapping-sensitive RA-MS responses in the rat. Although RA muscle afferents did project to areas near the forepaw and caudal lower lip, the majority of afferents projected to the darkly staining region connecting the forelimb and hindlimb representations (dashed lines). The random organization of the RA-MS unit responses is evident. In contrast, single electrode penetrations in the second somatosensory area (S2) had receptive fields that encompassed the entire forelimb.

We also recorded neural activity in the cortex using the degloved forelimb preparation and the identical stimulus paradigms for characterization of all the peripheral response subpopulations. We approached the brain mapping in two phases. In the first phase, the cutaneous nerves serving the glabrous skin of the forepaw were left intact to determine the general location of the cutaneous representation in the primary somatosensory area (S1) (Fig 4B–4H, red stars). In the second phase, the previously tagged nerves serving the forepaw were cut to silence all cutaneous input from the forelimb up to the level of the shoulder (Fig 4B–4H, black stars). During phase two mapping, when a cortical response to the tapping of the muscles on the degloved and distally denervated forelimb was isolated, we placed adjacent electrode penetrations more closely together until a non-responsive border or different modality border was found.

**Fig 5:**
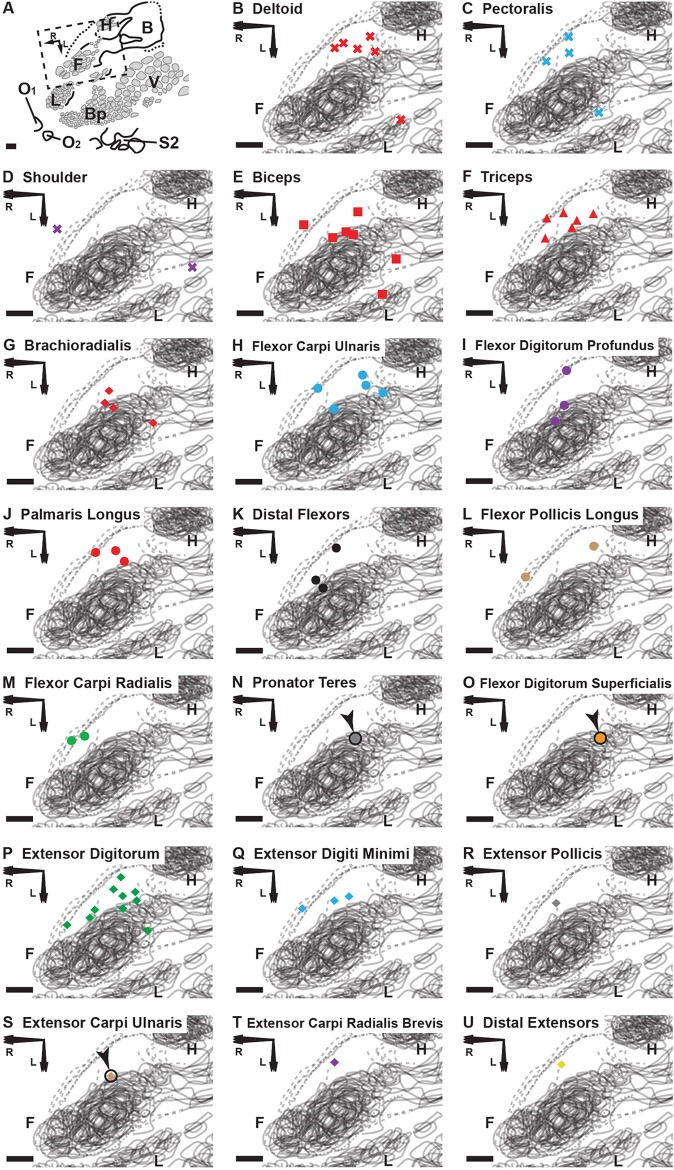
Cortical brain mapping of the RA-MS response group elicited by tapping the tendons of the degloved forelimb organized by individual muscle sensory response. **(A)** An overview of the left cortical hemisphere in the rat. Cytochrome oxidase delineated borders and barrel patterns of primary somatosensory cortex (black lines and grey shading): **F** = forepaw barrel subfield; **H** = hindpaw barrel subfield; **V** = vibrissae; **Bp** = buccal pad; **L** = lower lip, **O1** & **O2** = oral modules 1 & 2; **L** = chin/lower lip; **B** = body; **S2** = second somatosensory area. The dashed box denotes the area of interest enlarged in **(B)** through **(U)**. Scale bars = 500 μm; **R** = rostral; **L** = lateral. **(B-U)** The shapes of the labeled points refer to location or compartment: **X** = Shoulder; **Square** = Proximal Anterior Compartment; **Triangle** = Proximal Posterior Compartment; **Circle** = Distal Anterior Compartment; **Diamond** = Distal Posterior Compartment. We find that the muscles with the smallest overall representational area (the distal flexors and distal extensors) appear to project only to the transitional zone area that is rostral and medial to the S1 forepaw representation. In contrast, the proximal and middle muscles (except the triceps) and the extensor digitorum, each having a larger overall representational area, appear to project to transitional zone areas surrounding the S1 forepaw representation **(A-D, F and O)**. Note: Although not evident in the composite overlays, the receptive fields for the RA-MS-type response group are separate from S1 (Fig 4B–4H).

Following post-experimental alignment of the cortical electrophysiological and histological mapping data, we found that the responses from the RA-MS-type group were specifically clustered at the anterior-medial border of S1 between the forepaw and hindpaw representational regions (Fig 4I). Some penetrations also fell in the region between the caudal forelimb and lower lip/jaw/buccal pad barrel formations. However, all of the putative RA-MS-type group electrode penetrations, save a single response (Fig 4D), were entirely exclusive from the barrel structures of S1. This result is contrary to previous evidence by Gioanni (1987) that demonstrated muscle sensory responses to tendon pulling projecting across the entire cutaneous representation of S1 [43]. This difference could be due to the removal of all cutaneous afferents by degloving in this preparation versus pulling on the freed muscle tendons residing in situ under the native skin of the forelimb. The region where the RA-MS-type group responses were located, between the forepaw and hindpaw regions, stained more darkly than adjacent areas (Fig 4C–4F&4H, dashed lines). The organization of this RA-MS group response was only broadly topographic (Fig 4I). For example, the responses that projected to the forelimb were clustered mostly rostrally and around the S1 forepaw representation but did not extend into the territory near the S1 hindlimb representation. However, in clear contrast to the organizational properties of the S1 forepaw representation, there did not appear to be any stereotypic somatotopic placements for this response group within the cortex. Instead, the locations for the forelimb musculature were largely intermingled and varied considerably from animal to animal. There did not appear to be any specific evidence of borders or clustering based on function or compartment (Fig 5) and the typical barrel-type organizational structures that are visible in S1 cutaneous representation were not evident with cytochrome oxidase staining (Fig 4C–4H dashed lines). While no overt somatotopic organization was evident, the cortical receptive fields for the RA-MS-type group responses did appear to be myocentrically organized meaning that most electrode penetrations showed responsiveness to only a single muscle. Seventeen individual muscles of the forelimb were discretely represented in this small cortical area with short inter-electrode distances yielding entirely separate muscle receptive fields (Fig 4B–4H). For example, the smallest measured distance between penetrations representing distinct muscles was 128 μm (Fig 4D). In general, the average distance for the single closest penetrations from each case was 180 μm (SD = 50 μm, range = 128–279 μm). The average distance between a subset of 36 randomly chosen adjacent points across all 7 recording cases was 361 μm (SD = 187). Out of all 71 electrode penetrations in this region of cortex that were sensitive to forelimb muscle input only 5 showed response activity for more than a single muscle.

In 5 of the 7 experimental cases we also recorded the muscle sensory responses within the laterally-located second somatosensory area (S2) representation. The 10 recorded receptive fields for the afferents within S2 typically encompassed the entire forelimb (Fig 4I). In contrast, the receptive fields situated near S1 forepaw and hindpaw were discrete and muscle-specific.

### Cortical response properties

**Fig 6:**
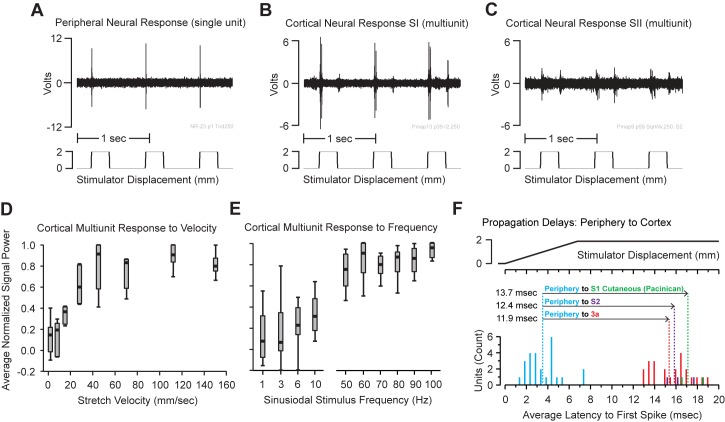
Peripheral and cortical response properties of the units searched out with tapping input. **(A)** A peripheral neural recording trace of an RA-MS-type single unit showing the characteristic responsiveness to the rapid onset of a square wave displacement and insensitivity to the static phase. **(B)** A cortical transitional zone multiunit (spike) recording of the input from a single forelimb muscle showing strong registration with the peripheral nerve activity. **(C)** A cortical S2 multiunit (spike) recording from an electrode penetration representing the entire forelimb showing similar response properties to both the peripheral and cortical transitional zone responses. **(D)** Calculation of cortical signal power shows an onset velocity of at least 30 mm/s was required for activation which was similar to the peripheral nerve RA onset rate dependent responses. **(E)** More cortical multiunit activity (increased signal power) was observed at the higher frequency band (50–100 Hz) with the highest measured activity recorded at 100 Hz (See also: Fig 2C and 2F). **(F)** Propagation delays from the periphery to cortex. Histograms for average time to first spike following a square wave input for 25 single units in the periphery (Blue), 23 single units in the transitional zone (Red), 3 single units in S2 (Purple), and 2 single units isolated with respect to a Pacinian corpuscle response in the cutaneous representation of S1 (Green). Average latencies for each population form the onset of the square wave were: 3.5 ms +/- 1.6 for the periphery, 15.4 ms +/- 1.7 for the transitional zone, 15.8 ms +/- 1.3 for S2, and 17.1 ms +/- 1.5 for the Pacinian response in the cutaneous representation of S1. The propagation delays from periphery to cortex were calculated by subtracting the average peripheral latency from the average cortical latencies for each cortical area.

When we were able to isolate clear multiunit (spike) cortical responses to a single muscle, we applied the identical manipulations and stimulus paradigms that were used in the peripheral recording experiments. We found a high-fidelity recapitulation of the RA peripheral activity in the cortex across both S1 and S2 (Fig 6A–6C). As with the peripheral RA-MS-type response group the cortical units were insensitive to prolonged static displacement of the muscles (Fig 6B). Measurement of cortical signal power associated with variable ramp speeds (without the frequency component) revealed that an onset velocity of at least 30 mm/s was required to activate a strong cortical response (Fig 6D) and, aligned well with the peripheral RA-MS-type group response profiles to vibration (as measured by single unit response) where the maximum slopes of the various frequencies equaled 31.4 mm/s at 100 Hz and 100 μm displacement, 39.3 mm/s at 50 Hz and 250 μm displacement, 78.5 mm/s at 50 Hz and 500 μm displacement, and 157.1 mm/s at 50 Hz and 1000 μm displacement (see: Fig 2C). Moreover, a measurement of cortical signal power in response to vibration revealed that all the cortical units were more active at the higher frequency band (50–100 Hz), with the highest measured signal power recorded at 100 Hz (Fig 6E) which also aligned well with the single unit spike counts seen in the peripheral recordings (see: Fig 2C). We calculated the propagation delays for a subset of individually identifiable (spike sorted) cortical units by taking the average time to first spike with a square wave displacement for recorded units at the anterior-medial border of S1 (15.4 ms +/- 1.7), a cutaneous Pacinian unit S1 (17.1 ms +/- 1.5), and units in S2 (15.8 ms +/- 1.2) and subtracted the onset delay from all the peripheral units recorded in the median nerve (3.5 ms +/-1.6) (Fig 6F). The propagation delays for the RA-MS-type group from periphery to cortex at the anterior-medial border of S1 and S2 were similar with the trip to S2 taking slightly longer; 11.9 ms vs. 12.4 ms, respectively (Fig 6F). During preliminary cortical mapping of the cutaneous responses in S1 we collected the latency to cortex for a specifically identified Pacinian corpuscle. The propagation delay for this mechanosensory unit at 13.7 ms was slightly longer (but not significant) than the RA-MS-type group propagation delays for the anterior-medial border of S1 and for S2.

**Fig 7:**
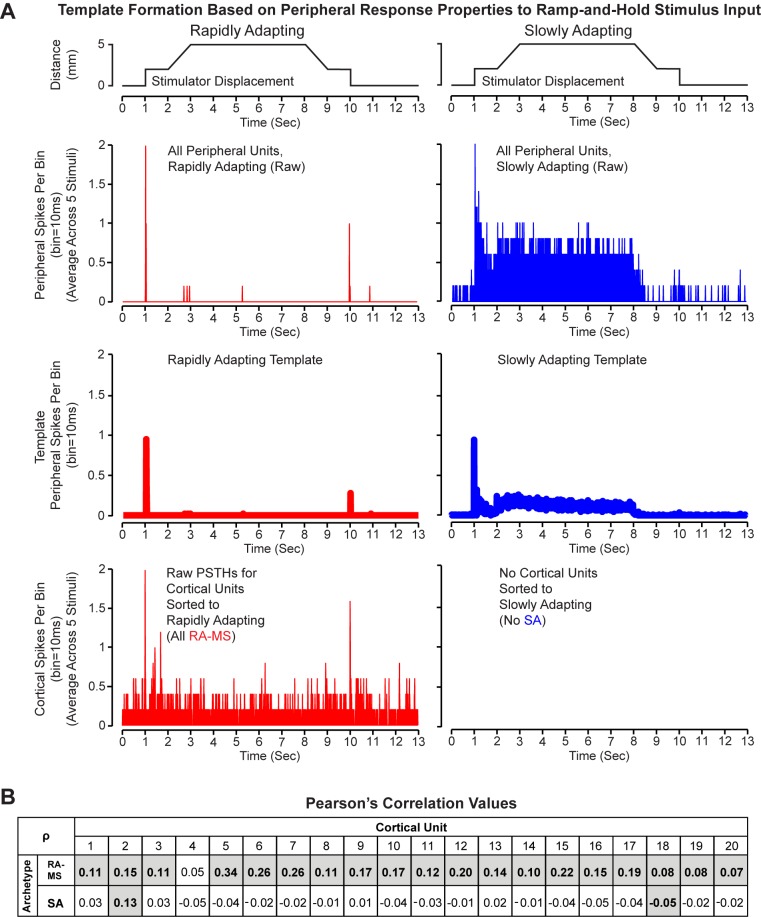
Response data from cortical units and peripheral units with application of ramp-and-hold stimuli. **(A)** Stimulator displacement (top), raw PSTH values for peripheral recordings from RA-MS-Type (red middle top) and SA units (blue middle top), template PSTH values for the RA-MS-Type (red middle bottom) and SA (blue middle bottom) peripheral response groups, and raw PSTH values for individual cortical units sorted to RA-MS (red bottom). All cortical recordings for ramp-and-hold inputs were classified to the RA-MS-type response group. **(B)** Matrix of Pearson’s correlation coefficients (ρ) calculated between each of the 20 individual cortical unit PSTHs (columns) and the two response group templates (rows). Values shaded with bold text indicate a significant correlation (p<0.05).

**Fig 8:**
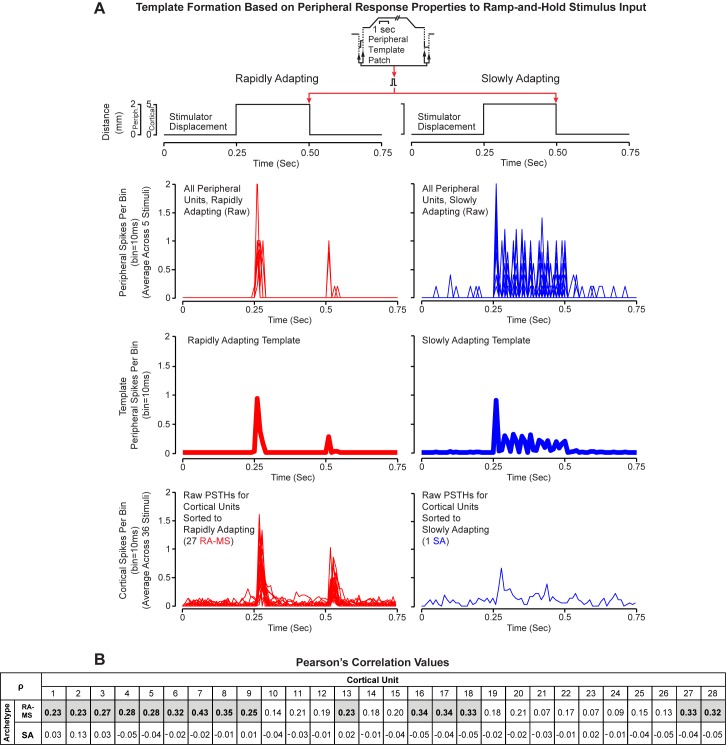
Response data from cortical units with application of square wave stimuli and from peripheral recordings patched from the square wave portions of the ramp-and-hold stimuli. **(A)** Stimulator displacement (top), raw PSTH values for peripheral recordings from RA-MS-Type (red middle top) and SA units (blue middle top), template PSTH values for the RA-MS-Type (red middle bottom) and SA (blue middle bottom) peripheral response groups, and raw PSTH values for individual cortical units sorted to RA-MS (red bottom) and SA (blue bottom). All but one cortical recording for square wave inputs were classified to the RA-MS-type response group. **(B)** Matrix of Pearson’s correlation coefficients (ρ) calculated between each of the 28 individual cortical unit transformed histograms (columns) and the two response group templates (rows). Values shaded with bold text indicate a significant correlation (p<0.05).

We collected cortical multiunit (spike) recordings for muscle sensory afferents in response to both ramp-and-hold inputs and square wave inputs. We investigated the response modes of these cortical units using PSTH-based classification of response profiles and Pearson’s correlations to determine the cortical PSTH similarity, with respect to timing and response magnitude for both the RA-MS-type and SA response groups in the periphery (Figs 7A and 8A). We found that the cortical units were more similar in response properties to the RA-MS-Type peripheral response group than they were to the SA (type Ia and type II) response group.

The PSTH-based classification demonstrated that all of the cortical units responded more like RA-MS-type than SA peripheral units to ramp-and-hold stimuli (Fig 7A; over the 13 s stimulation period, average Euclidean distances from RA-MS-type template 2.84+/-0.83, SA template 4.32+/-0.44 spikes per 10 ms bin) and all but one of the cortical units responded more like RA-MS-type than SA peripheral units to square wave stimuli (Fig 8A; over the 0.75 s stimulation period, average Euclidean distances from RA-MS-type classified cortical units to RA-MS-type template 1.41+/-0.30, SA template 1.68+/-0.29 spikes per 10 ms bin; SA classified cortical unit to RA-MS-type template 1.54, SA template 1.46 spikes per 10 ms bin). The Euclidean distance between the RA-MS-type and the SA templates was 3.57 spikes per 10 ms bin over the 13 s stimulation period during the ramp-and-hold stimulus and 1.01 spikes per 10 ms bin over the 0.75 s stimulation period during the square wave stimulus. While there is some overlap in response properties between the two templates at the step up and step down of both stimulus inputs, the behavior during the ramp portion and hold portions varies (Figs 7A and 8A, third row).

We also used Pearson’s correlations to compare the individual transformed cortical single unit PSTHs and the templates for each of the peripheral response groups. This complementary analysis showed that almost all of the cortical units were more positively associated with the RA-MS-type response group versus the SA response group for both the ramp-and-hold (all 20 cortical units) and square wave (27 of 28 cortical units) inputs (Figs 7B and 8B). Within the responses to the ramp-and-hold stimuli we found that 19 of the 20 cortical units showed a significant (p<0.02), though weak, positive correlation to the RA-MS-type group template with one also showing a significant (p<0.02), weak positive correlation with the SA group template (Fig 7B). Within the responses to the square wave stimuli we found that 15 of the 28 cortical units showed a significant (p<0.05) positive correlation to the RA-MS-type group template with none showing a significant positive correlation with the SA group template (Fig 8B).

## Discussion

In humans, the kinesthetic illusion elicits a strong joint-specific perception of limb movement although the limb is actually immobile [15, 20]. By using 70 Hz vibratory input as a search tool for evaluating muscle sensory responses we found a rapidly adapting group response that was most active within the same higher sinusoidal frequencies that elicit the kinesthetic illusion [20, 44] (Fig 2C and 2F). While there is no question that type Ia muscle spindle fibers are involved with proprioception, both the type Ia and type II muscle spindle populations showed a flat or lower-weighted response to all measured frequencies (Fig 2A, 2B, 2D and 2E). Similarly flat frequency response profile results for type 1a, type II, and Golgi tendon organs have been demonstrated with human single unit microneurography as well [45]. Importantly, the type Ia muscle spindles, which are considered to be the receptor responsible for the kinesthetic illusion, were equally highly sensitive to all of the measured vibratory frequency responses across 1–10 Hz and 50–100 Hz (Fig 2A and 2D). It is unlikely that a flat frequency tuning would encode for the kinesthetic illusion that is most highly active from 70–115 Hz [20, 44]. This is particularly compelling as a similarly broad frequency response profile is described in human microneurography work that implicates the type Ia muscle spindle fiber as the likely mediator of the kinesthetic illusion [15]. It is important to note that the presence of anesthesia may have adverse effects on the response behaviors of these receptors. Urethane was chosen because it is typically used for cortical mapping studies, but it is known to depress muscle spindle activity [46].

The results presented here appear to provide evidence that the RA-MS-type peripheral and cortical response groups may not be directly attributed to muscle spindles, and further work should be undertaken to identify the sensory receptor subtype that is contributing to the RA-MS response group. There are many specialized sensory receptor organs located in skeletal muscles [47]. Although the RA-MS channel appeared to respond differently from the type Ia or type II muscle spindle fibers demonstrated here, activity from other receptor channels, such as Golgi tendon organs and Pacinian corpuscles, must be taken into account.

Golgi tendon organs are the most likely source of the RA-MS response group. Similarly to the RA-MS group described here, Golgi tendon organs exhibit rapidly adapting responses that are sensitive to tapping in the relaxed muscle [48] but insensitive to static displacement and the dynamics of ramp-and-hold inputs [8, 10]. Golgi tendon organs respond to rapid onset stimuli with high instantaneous frequency bursts of multiple action potentials [49]. Although, in this preparation we found that the RA-MS units fired a single (occasionally a second) impulse at the rapid onset of a square wave displacement. Golgi tendon organs will occasionally fire single impulses at the onset of a slow ramp stretch [8]; however, no such impulses were evident in our recordings. Others have proposed that the initial burst of activity at stimulus onset for a Golgi tendon organ is due to stuck actomyosin bonds [10, 48, 49] and that they do not track well to various sinusoidal inputs. In contrast, with this preparation in relaxed, uncontracted, muscle we found that the RA-MS response group units reliably followed sinusoidal input at 1:1 for extended periods; up to 3600 cycles over multiple displacements and frequencies (Fig 2F). This suggests that sustained high-fidelity firing is likely a property of the response group and not an artifact of stuck actomyosin bonds. This is particularly interesting because in work by Fallon and Macefield, using a human single unit microneurography preparation specifically designed to maximize isolation of Golgi tendon organs, it was found that they do not react to vibratory input in relaxed, uncontracted, muscle [45]. In the same study it was also found that when a contraction was generated by the participants the Golgi tendon organs did respond strongly to vibration but no preferred frequency for these units was demonstrated. In our work here we found that bypassing the myotendinous junction, where Golgi tendon organs reside, still resulted in activation of the RA-MS type responses suggesting a possible location for this response group is within the belly of the muscle (Fig 3). In all, these differences in findings suggest that further investigation to identify the RA-MS response group observed here is required. For example, since these experiments were undertaken under conditions of passive stretch monitoring the RA-MS response group during active muscle contractions may further help to confirm or rule out its identity as a Golgi tendon organ, and monitoring activity in response to an injection of succinylcholine to confirm or rule out its identity as a primary muscle spindle.

Pacinian corpuscles are responsive to dynamic phase changes in square wave input and tuned to vibration (100–250 Hz) that overlaps with the frequency response of the RA-MS units [50, 51]. However, the response group demonstrated here does not track dynamic phase changes; instead it typically fires a single impulse at the onset of stimulus and occasionally on the release of the static stimulus (Fig 1D).

The RA-MS response group described here was inactive at the lower non-illusionary frequencies, yet well-tuned to the 70Hz and higher frequencies that elicit the kinesthetic illusion [20, 21, 44]. Interestingly, when skeletal muscle motor units contract they vibrate strongly enough to generate measurable sounds [52]. A 5^th^ order visco-elastic mechanical system model, based on phonomyography and electromyogram spike-triggered averaging, suggests that single motor units longitudinally resonate at 93 and 166 Hz when contracting [53]. These resonant frequencies overlap the ranges that trigger the kinesthetic illusion and match well to the most active frequency response (90 Hz) of the RA-MS-type response group demonstrated here (Figs 2C and 6D). Therefore, this RA-MS-type response group may be implicated in contributing to encoding a kinesthetic sense and could represent a neural system to pick up the intrinsic vibration of active muscle contraction to perceive movement which can also be triggered by externally applied vibration to elicit the kinesthetic illusion.

The muscle sensory units that were recorded in the cortex appeared to present with a response mode that was different than the slowly adapting type 1a and type II units that were identified in the peripheral recordings. We compared the activity profiles of the cortical units to the activity profiles of the peripheral rapidly adapting and slowly adapting muscle sensory populations in response to both ramp-and-hold and square wave displacements. Using two complementary approaches (PSTH-based response classification and Pearson’s correlations) we found a clear similarity between the activity profiles of the cortical units and the RA-MS-type group response in the periphery. Using PSTH-based response classification (Figs 7A and 8A) we separated the peripheral RA-MS-type response group units from the peripheral SA group units. We found that the individual units recorded in the cortex reflected a response mode that was different than the slowly adapting activity of the peripheral type Ia and type II muscle spindles. All of the individual cortical units classified into the RA-MS group response category when stimulated with ramp-and-hold input (Fig 7C) while all but one of the individual cortical units classified into the RA-MS group response category when stimulated with square wave input (Fig 8C). The majority of the cortical units in response to both ramp-and-hold and square wave inputs showed greater positive associations (Pearson’s correlation: rho) with the peripheral RA-MS-type response group template than with the peripheral SA group template (Figs 7B and 8B). There were significant correlations between peripheral RA-MS-type response group template and the cortical units; 19/20 of the cortical units within the ramp-and-holds (Fig 7B) and 15/28 cortical units within the square waves (Fig 8B).

In primates it has been demonstrated that muscle sensory afferents project primarily to cortical area 3a whereas cutaneous tactile afferents project widely across primary somatosensory cortex (areas 3b, 1, and 2) and also to area 3a [54]. Evidence suggests that central processing of tactile input is likely based on convergence and integration of relevant features across multiple receptor submodalities [55]. Similarly, differences between cortical and peripheral proprioceptive response properties are typically attributed to convergent activation from tactile afferents or from additional processing within relays along the pathway to the cortex [4, 56]. Removal of the cutaneous afferents due to the de-gloving procedure may have helped to expose the RA-MS cortical group response. Without cutaneous mechanoreceptive input it is unlikely that the RA-MS response profiles observed here were produced solely as a product of convergent modification from tactile afferents in the transitional zone from the cutaneous representation in S1. For example, we had clear evidence that cutaneous mechanosensory afferents projected to the region anterior to S1 from the recordings taken prior to nerve section at the wrist during the first phase of cortical mapping (see: red stars Fig 4B–4D and 4F–4H). However, these tactile afferent responses were reliably silenced after the combined forepaw denervation and degloving (see: black X’s Fig 4B–4H). The propagation delay from the periphery to the cutaneous representation of S1 cortex for a response clearly identified as an unprocessed Pacinian corpuscle isolated during the cutaneous phase of cortical mapping was 13.7 ms. This was slightly slower than the average propagation delay for the cortical RA-MS group afferents that arrived in the transitional zone anterior to the S1 cutaneous representation at an average of 11.9 ms from the periphery (Fig 6F). Somatosensory evoked potentials in primates have revealed that convergent tactile input from the periphery into 3a takes longer than a direct connection from the muscle sensory afferents [54]. The commensurate times for the afferent responses to primary cortex that we show here provide evidence that the RA-MS-type group response is likely reflective of a direct communication between the periphery and primary sensory cortex. It is also important to note that in the mechanosensory system that signals appear to arrive in cortical layers 4/5 (the recording depth reflected in this study) from the periphery largely intact with respect to overall response properties [57, 58]. Furthermore, similar response profiles and propagation delays (12.4 ms) were identified for the RA-MS response group recorded in S2 as well (see: Fig 6C and 6F).

Additional evidence points to the involvement of this RA-MS-type group response with processing of kinesthetic sensation. We found that their stimulation elicited activity in the cortex that clustered within a darkly staining region between forepaw and hindpaw areas at the anterior-medial border of the somatotopic cutaneous representation of S1 (Fig 4C–4H, dashed lines). Some of these afferents also projected to the posterior-lateral border of the S1 forepaw/forelimb (Fig 4C and 4E). These regions of the rat cortex rostral to and surrounding the granular S1 forepaw representation have been characterized as the “transitional zone” [59] and the “lateral agranular field” with “S1 dysgranular cortex” [60]. In the ground squirrel they have been characterized as the “rostral field (R)” [61] and as “area 3a” [62]. This area of the brain has also been identified as the “kinesthetic cortex” in carnivores [63]. Based on functional organization, connectivity, myeloarchitecture, and similarities between other mammalian brain areas, this region of cortex is considered to be the rodent homolog of cortical area 3a, a region known to process proprioceptive input [61, 62]. Furthermore, this RA-MS-type group also showed receptive fields in the lateral second somatosensory area (S2), a brain region with a prominent role in proprioceptive sensory-motor integration and immediate post-movement processing of voluntary actions [64–66]. In this cortical association area the RA-MS-type group receptive fields were much larger and encompassed the entire forelimb instead of being muscle-specific as in the region adjacent to S1 (Fig 4I).

The evidence for the myocentric organization found in this study is compelling because it has been suggested that the brain likely accesses muscle-specific kinesthetic information [67]. For example, in humans, vibration of a single muscle can generate an illusionary sensation of joint movement [67]; whereas simultaneous illusionary stimulation to antagonistic muscles abolishes the perception of limb movement [15]. This is interesting because we found seventeen muscles of the forelimb discretely cortically represented with small inter-electrode distances often yielding entirely separate individual muscle receptive fields at each penetration (Fig 4B–4H).

Moreover, muscle specificity, not topographic fidelity, appeared to be the primary organizational property of this representational area. The receptive fields for the RA-MS-type group showed only a coarse topography where the responses that projected to the forelimb were clustered around the S1 forepaw representation but did not extend into the territory near the S1 hindlimb (Fig 4I). Otherwise, the forelimb musculature receptive fields were variable between animals and intermingled within each animal with no evidence of borders or clustering based on topographic location, function, or compartment (Fig 5). This has also been reported in a population of deep muscle sensory afferents that were sensitive to tapping within primate cortical area 3a in marmoset monkeys [68].

**Fig 9:**
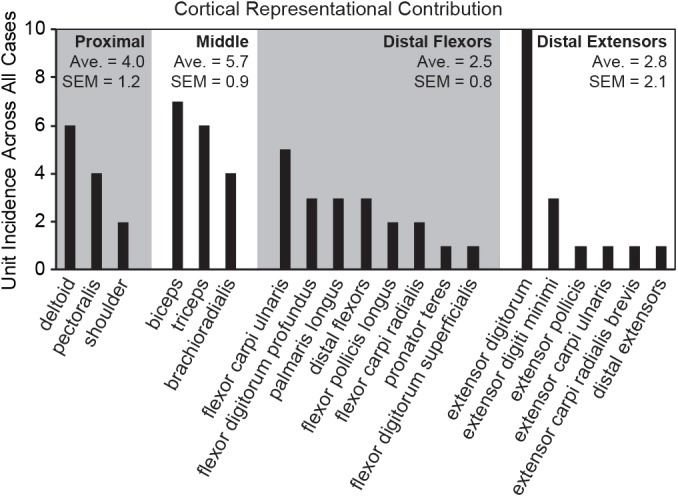
A count of the incidence of each muscle receptive field recorded in the cortex across all seven brain mapping cases. The highest number of RA-MS group responses projected to muscles crossing the proximal joints and the extensor digitorum, with fewer responses projecting to the distal phalangeal flexors and extensors.

Proximity to the midline and multi-articulation may be additional organizational properties of this cortical region. Larger cortical representational areas confer increased processing resources. Tactile hyperacuity of individual fingertips in humans is determined by the size of the cortical representation in primary somatosensory cortex, and the fovea of the eye has a higher visual acuity than would be predicted by receptor density alone [69, 70]. A count of individual muscles represented across all of the randomly placed tapping-sensitive electrode penetrations revealed a larger number of the muscles crossing the proximal limb joints (deltoid, pectoralis, biceps, triceps and brachioradialis) than those crossing the distal digit joints (digit flexors and extensors) with the largest contribution from the extensor digitorum (Fig 9). This preponderance of shoulder and elbow movers and specifically a wrist extensor is also evident in microstimulation studies of the rodent M1-S1 transitional zone [60, 71–73]. Humans are more sensitive to movement at the proximal limb joints than they are at the distal joints [74–76]; however, when sensitivities to muscle length change are measured proportionally to overall muscle length it shows that the muscles themselves are equally sensitive to movement regardless of location on the proximal-distal axis [74]. With similar peripheral sensitivities across the proximal-distal axis it is suggestive that higher-order processing may be involved with increased movement sensitivity at the proximal joints. For example, when pointing, a small movement at the shoulder produces a large positional movement of a pointing finger whereas a small movement of the finger itself produces a much smaller positional movement. The large representation of muscles prone to large positional movement changes and control across multiple joints may suggest a role in error processing [77]. In all, these results suggest further avenues of investigation into the specific peripheral receptors and the representational organizational structures that are responsible for kinesthetic perception.

Kinesthesia is integral to motor control and normal movement. Rehabilitation strategies may benefit from approaches that focus on the possible muscle-specific error calculation and representational processing along the proximal-distal axis with direct implications for physical therapy following stroke and spinal cord injury. A better mechanistic understanding of kinesthesia may provide for refined approaches for receptor-specific peripheral nerve stimulation for sensory feedback in prosthetic limbs and for direct cortical feedback in brain-machine-interfaces. Beyond the clinic, insight into the functional organizational properties of movement feedback in an evolved system provides fertile ground for optimizing machine control, robotics and cobotics. Our understanding of perception and cognition as a whole may be impacted by a better understanding of the mechanistic underpinnings of proprioception.
